# Case Report of Successful Childbearing after Conservative Surgery for Cervical Mullerian Adenosarcoma

**DOI:** 10.1155/2017/4187416

**Published:** 2017-01-05

**Authors:** Seiji Kanayama, Masako Nakamura, Hidekazu Oi, Sumire Sugimoto, Yoshikazu Sasaki, Tomoko Uchiyama, Chiho Ohbayashi, Hiroshi Kobayashi

**Affiliations:** ^1^Department of Obstetrics and Gynecology, Kinki University Nara Hospital, 1248-1 Otuda-Chou, Ikoma, Nara 630-0293, Japan; ^2^Department of Obstetrics and Gynecology, Nara Medical University, 840 Shijo-cho, Kashihara, Nara 634-8522, Japan; ^3^Department of Diagnostic Pathology, Nara Medical University, 840 Shijo-cho, Kashihara, Nara 634-8522, Japan

## Abstract

Mullerian adenosarcoma (MA) is a rare tumor variant with low malignancy potential and is reported to account for 8% of all uterine sarcomas. Cervical MAs are reported to occur in relatively younger patients with the mean age of 27 years, while those in the uterine corpus generally present in postmenopausal women. Due to the rarity of cervical MAs, optimal management for these patients (especially younger women) is still under exploration. Here, we describe a case of cervical MA in a woman of reproductive age who was treated by fertility-preserving surgery and successfully delivered a child 18 months later.

## 1. Introduction

Mullerian adenosarcoma (MA) is a rare tumor with usually low malignancy potential and has been reported to account for 8% of all uterine sarcomas. MAs are composed of two different components: sarcomatous stromal lesions (usually of the endometrial low-grade stromal type) and benign or mildly atypical epithelial elements [1–3]. The presence of one or more of the following criteria results in a diagnosis of MA: a stromal mitotic count of 2 or more mitotic figures/10 HPF, marked stromal cellularity, and mild or moderate nuclear atypia of the stromal cells [4]. The most frequent primary site of MA is the uterine endometrium, but these tumors can also arise (albeit rarely) in uterine cervix, ovary, vagina, and fallopian tubes.

Cervical MAs are reported to account for 2% to 9% of all MA cases [2, 4, 5]. Clinical presentation of cervical MAs often resembles either a benign endocervical or endometrial polyp or a pedunculated submucosal leiomyoma protruding through a cervical canal. Cervical MAs are reported to occur in relatively younger women (mean age 27 years), while tumors originating from the uterine endometrium generally present in postmenopausal women (median age 58 years) [4, 6].

Due to the rarity of cervical MA, there are limited data concerning its prognosis and management. Therefore, optimal management for the cervical MAs in younger women continues to be explored. Here, we present a case of a woman of reproductive age diagnosed with cervical MA and treated by fertility-preserving surgery; she successfully delivered a child 18 months after receiving the initial treatments.

## 2. Case Presentation

**Figure 1:**
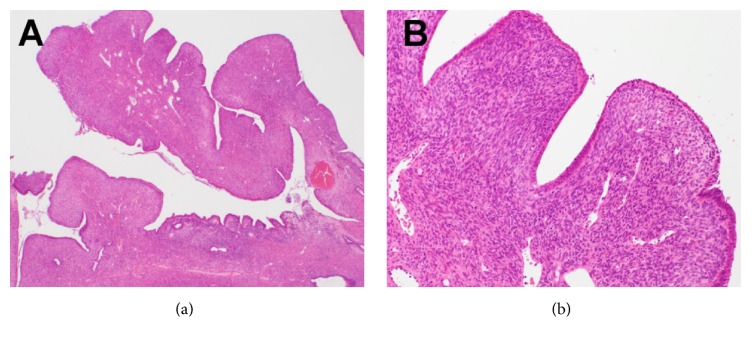
(a) Tumor cells with two components: endocervical glandular lesion without atypia of a phyllodes-like pattern and periglandular, spindle stromal cell condensation. (b) Stromal spindle cells with mild nuclear atypia. The mitotic counts were greater than two mitotic figures/10 HPF.

A 28-year-old healthy nulliparous woman presented with a short history of metrorrhagia of 30-day duration. Previous medical history was unremarkable. Pelvic examination revealed a 4 × 5 cm friable cervical mass with an irregular and hemorrhagic surface that appeared to be protruding through the cervical ostium. Transvaginal ultrasonography showed a normal shape of the uterine corpus and a smooth endometrium. The tumor was resected for biopsy. Macroscopically, the tumor presented as an irregular polypoid mass with granular and papillary surface. Upon microscopic examination, the tumor comprised two components: endocervical glandular lesions without atypia of a phyllodes-like pattern and a periglandular, cambium-like layer of spindle stromal cell condensation with mild nuclear atypia (Figure 1). The mitotic counts were more than two mitotic figures/10 HPF. The mesenchymal lesion showed neither sarcomatous overgrowth nor heterologous elements such as cartilage or rhabdomyosarcoma. The tumor was pathologically diagnosed as Mullerian endocervical heterologous adenosarcoma. MRI of the pelvis showed no residual uterine tumor and significant pelvic lymphadenopathy. Tumor markers such as CA125, CA19-9, and CEA were all within normal ranges. Then, she underwent conization of the uterine cervix to confirm the existence of a residual lesion. Pathological diagnosis of the conization showed mainly normal cervical epithelium with no residual sarcomatous lesion.

**Figure 2:**
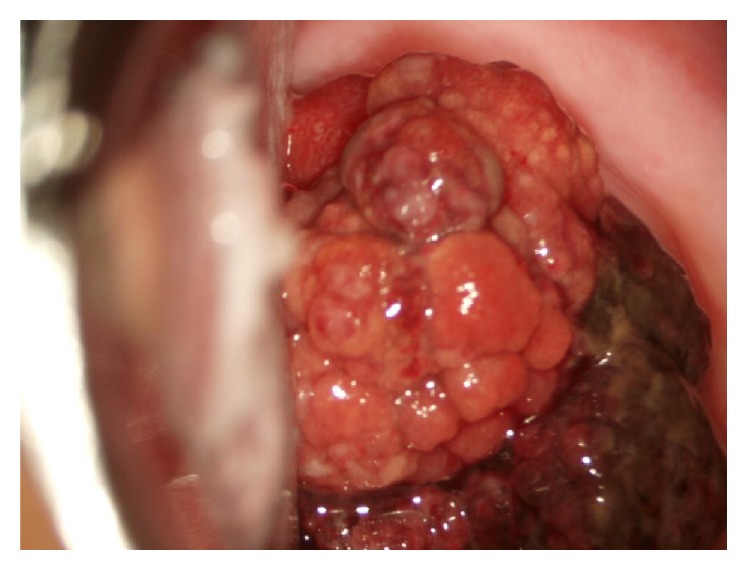
Recurrent polypoid and hemorrhagic irregular tumors filling the vagina and protruding through a cervical canal.

Although she received sufficient explanation and fully understood the risk of recurrent disease, the patient and her husband desired to preserve their fertility. Therefore, she wished to have monthly follow-up visits for careful surveillance to avoid hysterectomy or hormonal therapy. However, 3 months later, she presented recurrent 4 cm polypoid and hemorrhagic irregular tumors filling the vagina from thin stalk (Figure 2). She underwent transcervical resection (TCR) to preserve her fertility. The pathological diagnosis after TCR showed focal sarcomatous remnants without involvement of the tumor stalk and resembled findings from the first biopsy. Endometrial curettage showed no remaining tumor. After continuous and careful monthly follow-up, she successfully conceived naturally and delivered a healthy infant 18 months after surgery. She is alive with no evidence of disease 32 months after TCR.

## 3. Discussion

MAs are a rare tumor variant with low malignancy potential and have been reported to account for 8% of all uterine sarcomas. Typical symptoms of cervical MAs include abnormal genital bleeding accompanied by polypoid lesions protruding through the external cervical os. The initial impression of these tumors resembles benign cervical polyps. Therefore, some cases are initially diagnosed as a benign cervical polyp [7] due to the rare incidence of cervical MAs. A history of recurrent cervical polyps is clinically important when suspecting the possibility of MA.

The etiology of MA is still unknown due to the rarity of these tumors. Some literature reported that a history of long-term use of oral contraceptives or tamoxifen might possibly be associated with the development of MA [3, 8–10], although, in our case, the patient had no history of any of these factors. Clement and Scully reported in a series of 100 cases that the recurrence rate after surgery was 23.9% [4].

Unfavorable factors for cervical MAs include sarcomatous overgrowth, necrosis, cytologic atypia, high mitotic rate, heterologous elements, deep myometrial invasion, and extrauterine spread [7, 11]. Of these features, sarcomatous overgrowth and deep myometrial invasion are thought to be the most important adverse prognostic factor [6, 12, 13]. MAs with sarcomatous overgrowth are reported in approximately 33% of uterine corpus MAs and approximately 60% of those cases relapsed [1]. Fortunately, in our case, the patient had no poor prognostic factor.

The prognosis of cervical MA is unknown due to its low incidence and the absence of long-term follow-up data. Previous studies have reported that cervical MA seemed to trend towards relatively slow progression. In a series of 100 cases, Clement and Scully reported that the recurrence rate of MA after surgery was 23.9% and that one-third of the recurrences occur after 5 years [4]. Therefore, long-term follow-up longer than 5 years is thought to be necessary for adequate surveillance.

There is no consensus on the optimal management of cervical MA. Many authors have recommended total hysterectomy with bilateral salpingo-oophorectomy as a curative treatment. The necessity of lymphadenectomy is unknown due to the low incidence of pelvic (6.5%) and para-aortic (0%) lymph node metastasis [8].

MAs are low-grade neoplasms and are capable of local recurrence after polypectomy or hysterectomy and, much less commonly, distant metastasis. The reported local and distant recurrence rates are 24% and 2%, respectively, after hysterectomy and bilateral salpingo-oophorectomy [4].

Cervical MAs have been reported to occur in relatively younger women (mean age 27 years) [6]. On the other hand, uterine MAs generally present in postmenopausal women (median age 58 years) [4]. In the review by Ramos et al., one-third of patients were younger than 15 years old [13].

In many cases, the strategy for the treatment of cervical MA is based on experience with uterine MAs. Therefore, therapeutic options for cervical MA patients who desire to preserve their fertility are still unknown. Until now, there have been only rare cases of women who desire fertility-preserving surgery and undergo successful treatment via local excision [4, 13–15]. These reports suggest that fertility-preserving surgery may be an alternative for patients with pedunculated cervical polypoid tumors with disease-free stalks [5, 16]. However, there is inadequate evidence to support either local resection or ovarian preservation for younger patients.

To date, there have been only two reports describing successful pregnancies in patients with MA who underwent local resection. Chin et al. reported, that among their 9 patients with cervical MAs, one 17-year-old patient who underwent cervical wedge resection successfully conceived and delivered a healthy infant after surgery and remained disease-free throughout the 204-month follow-up [15]. Zaloudek and Norris reported that, among 35 patients, two teenage girls were treated by wide local excision of cervical adenosarcoma and remained well for the follow-up duration; furthermore, one of them successfully conceived after treatment [14]. To the best of our knowledge, our present patient is likely the third reported case in the English literature.

The risk of ovarian metastasis among patients with uterine MA is relatively low, as initial ovarian involvement is reported to be approximately 2% [4, 17].

Thus, Michener and Simon reported that ovarian conservation can probably be safely achieved in carefully selected women with MA who are of reproductive age.

On the other hand, recurrence rates for patients undergoing local resection were reported to be as high as 50% compared to approximately 25% for patients treated by hysterectomy [4]. Fleming et al. reported that, in 12 patients with cervical MA, 5 patients underwent fertility-preserving surgery, 4 patients underwent either D&C or polypectomy, and 1 patient underwent trachelectomy. Among the 5 patients who underwent surgery, recurrence occurred in 4 of them, and the recurrence sites were all in endometrium with a time until recurrence ranging from 3 to 11 years [18].

## 4. Conclusion

Here, we present a patient of reproductive age who was diagnosed with cervical MA and treated by fertility-preserving surgery; this patient successfully delivered a child 18 months after her initial treatment and experienced no recurrent disease. However, previous several case reports demonstrated high recurrence rates for patients undergoing local resection compared to patients treated by hysterectomy. So we should still be cautious as much as possible when offering a patient fertility-preserving surgery, as there has been inadequate evidence to support the use of local resection or ovarian preservation for younger patients. Furthermore, an accumulation of case reports concerning the long-term follow-up and obstetrical outcomes after fertility-preserving surgery is needed to establish optimal management for these patients.
